# Gene Copy Number Variation Does Not Reflect Structure or Environmental Selection in Two Recently Diverged California Populations of *Suillus brevipes*

**DOI:** 10.1534/g3.120.401735

**Published:** 2020-10-13

**Authors:** Anna L. Bazzicalupo, Mallory Thomas, Robert Mason, Munro-Ehrlich, Sara Branco

**Affiliations:** * Department of Microbiology and Immunology, Montana State University, Bozeman, MT; † Department of Integrative Biology, University of Colorado Denver, Denver, CO

**Keywords:** fungi, copy-number variants, Suillus, salt tolerance, mycorrhiza

## Abstract

Gene copy number variation across individuals has been shown to track population structure and be a source of adaptive genetic variation with significant fitness impacts. In this study, we report opposite results for both predictions based on the analysis of gene copy number variants (CNVs) of *Suillus brevipes*, a mycorrhizal fungus adapted to coastal and montane habitats in California. In order to assess whether gene copy number variation mirrored population structure and selection in this species, we investigated two previously studied locally adapted populations showing a highly differentiated genomic region encompassing a gene predicted to confer salt tolerance. In addition, we examined whether copy number in the genes related to salt homeostasis was differentiated between the two populations. Although we found many instances of CNV regions across the genomes of *S. brevipes* individuals, we also found CNVs did not recover population structure and known salt-tolerance-related genes were not under selection across the coastal population. Our results contrast with predictions of CNVs matching single-nucleotide polymorphism divergence and showed CNVs of genes for salt homeostasis are not under selection in *S. brevipes*.

Copy-number variants (CNVs) are genes or genomic regions of varying size with different numbers of copies across individuals. Copy-number differences can arise through genomic rearrangements (such as non-allelic homologous recombination and non-homologous end-joining (Gu *et al.* 2008; Conrad *et al.* 2010; Lupski and Stankiewicz 2005), stalled replication forks (Hull *et al.* 2017), and DNA circularization (Møller *et al.* 2015). CNVs can be population-specific and have a large impact on population dynamics. For example, selection has been shown to efficiently remove harmful CNVs in large populations of malaria parasites and less efficiently in smaller populations (Cheeseman *et al.* 2015). Population structure and selection histories can be recovered in population-level analyses of CNVs. In humans, CNVs were shown to reflect the population structure detected from single-nucleotide polymorphisms (SNPs) divergence (Jakobsson *et al.* 2008; Conrad *et al.* 2010; Redon *et al.* 2006). Even though CNVs mirror population structure patterns recovered from single-nucleotide polymorphisms (SNPs) in many organisms including humans, in the grass *Brachipodium distachyon* (Gordon *et al.* 2017), in fungal pathogens *Zymoseptoria tritici* and *Aspergillus fumigatus* (Hartmann and Croll 2017; Zhao and Gibbons 2018), in high-altitude adapted Chinese indigenous cattle (Zhang *et al.* 2020), this is not necessarily always the case. For example, a study on an American lobster population across an inlet temperature gradient showing no population structure based on SNPs found CNVs to be better predictors of selection than SNPs and a mismatch between the pattern recovered by SNPs and CNVs (Dorant *et al.* 2020).

Discovering CNVs across individuals is important because variants causing drastic changes in phenotypes can be adaptive and have major evolutionary consequences. Selection can modulate protein dosage (where increased copy-number leads to increased amounts of gene product in the cell) or produce functional divergence across gene copies resulting in differentially adaptive phenotypes (Schrider and Hahn 2010; Kondrashov 2012). CNVs, and in particular gene multiplications, are known to be important in environmental adaptation (Oh *et al.* 2012). Gene copy number variation is known to be involved in adaptation to environments characterized by extreme temperatures, toxic heavy metals, and high salinity (Kondrashov 2012; Oh *et al.* 2012). For example, the expansion of the antifreeze glycoprotein gene family allows cod to withstand freezing temperatures in the Antarctic (Chen *et al.* 2008) and increased numbers in heat-tolerance related gene copies confer heat tolerance in strains of *Escherichia coli* (Riehle *et al.* 2001). The evolution of tolerance to heavy metals can also occur through CNVs (Oh *et al.* 2012), including increased number of copies and expression levels of metal homeostasis genes in plants (Craciun *et al.* 2012; Talke *et al.* 2006) and fungi (Ruytinx *et al.* 2019; Bazzicalupo *et al.* 2020). Salt tolerance can also be associated with ion transporter gene CNVs and has been documented in several plants (Huang *et al.* 2008; Wu *et al.* 2012) and in the yeast *Saccharomyces cerevisiae*, where increased levels of ploidy and expression are associated with tolerance to high salinity (Dhar *et al.* 2011).

Fungi lack easily measurable phenotypes, making studying fungal environmental adaptation challenging (Branco *et al.* 2017; Branco 2019). One common approach to detect genomic signatures of selection is to use both genome scans for selection and correlations between genomes and environment, underlying the importance of specific genes in the ability of fungi to persist in specific environments. For the most part, this approach has been most often applied to SNP data (Ellison *et al.* 2011; Gladieux *et al.* 2015; Branco *et al.* 2015; Branco *et al.* 2017). However, given the evolutionary importance of CNVs, genome scans targeting copy number variation have also been useful in detecting adaptive variants in fungi such as wine strains of the yeast *Saccharomyces cerevisiae*, the human pathogen *Cryptococcus gattii*, and the mycorrhizal fungus *Suillus luteus* (Steenwyk and Rokas 2017; Steenwyk and Rokas 2018; Steenwyk *et al.* 2016; Bazzicalupo *et al.* 2020).

Here, we investigate whether gene copy number variation mirrors the patterns of population structure and selection unveiled by genome scans on SNP divergence in *Suillus brevipes*, a widespread mycorrhizal fungus associated with pines. Previous work documented differentiated populations from coastal and montane regions in California adapted to distinct climatic and salinity conditions (Branco *et al.* 2015; Branco *et al.* 2017), with SNP differentiation revealing a strong signature of selection near a salt-tolerance related gene (predicted as *Nha-1*-like, an Na^+^/H^+^ antiporter). Furthermore, no SNP variation was found in this gene across coastal individuals strongly suggesting a selective sweep for adaptive alleles allowing colonization of saline environments in coastal California. *Nha-1* homologs have been reported as important for salt tolerance in other organisms, including increased *Nha-1* expression levels in *Arabidopsis thaliana* allowing survival in high sodium concentrations (Apse *et al.* 1999) and *Nha-1* mutations conveying salt tolerance in yeast (Nass *et al.* 1997). In addition, there is evidence for salt tolerant yeast strains having multiple copies of *Nha-1* (Prior *et al.* 1996). We hypothesized that CNVs in the two *S. brevipes* populations mirror both population structure and selection patterns recovered from SNP analyses, and are prevalent in specific genomic regions related to salt homeostasis. Specifically, we investigated whether CNVs recover the previously described population differentiation between coastal and montane California and if the most differentiated CNVs involved salt homeostasis genes, including the *Nha-1*-like gene. We found fungal individuals to show many CNVs, however, these CNVs failed to recapitulate the *S. brevipes* population structure and signatures of selection predicted by SNP analyses.

## Methods

### Gene copy-number estimates per individual and population gene copy-number differentiation

We estimated gene copy number variation from the processed Illumina reads of 27 *S. brevipes* whole genomes from coastal (11 individuals) and montane (16 individuals) California populations previously analyzed in Branco *et al.* (2015). Read data are available in GenBank (see Branco *et al.* (2015) for SRA codes of individuals and reference genome). To detect copy-number variation across populations, we estimated the number of copies for 250 base pair windows in each individual using the software Control-FreeC (Barillot *et al.* 2011). *Suillus brevipes* is a dikaryotic fungus which means two haploid genomes inhabit the same cell, we therefore assumed diploidy of our samples and followed Steenwyk *et al.* (2016) for other software settings. Control-FreeC aligns the reads to the reference genome, normalizes them assuming diploidy, and estimates deviations based on the read count. Only genes present in the reference are estimated for copy-number deviations and genes absent from the sample are counted as losses. We define ‘gains’ as regions estimated to have more than two copies and ‘losses’ or ‘absences’ as regions that are estimated to have one or zero copies of a gene. This method based on normalized read counts does not allow us to truly assess if the copies of a particular gene are found in the same genomic locations or other parts of the genome. To identify copy number differentiated regions we calculated the amount of copy variance using V_ST_ following Steenwyk *et al.* (2016) (code with V_ST_ formula is available from https://github.com/abazzical/sbrevCNV). Given the reference genome is rather fragmented (Branco *et al.* 2015), we report results for only the 100 largest scaffolds of the genome for all subsequent analyses. The total reference genome length is 52,222,250 bp and scaffolds 1-100 represent 54.7% (28,583,750 bp). As scaffolds in reference genomes are numbered according to their size, we observed an increase in the V_ST_ values as the size of the scaffold decreased possibly indicating some systematic error in copy number estimate when the reference scaffold is more fragmented. We therefore decided to only use the first 100 scaffolds (Fig S1-2).

### Clustering individuals based on whole-genome copy-number variants (CNVs)

To investigate whether copy number variation recovered the population differentiation found in (Branco *et al.* 2017; Branco *et al.* 2015), we performed Principal Coordinates Analyses (PCoA) based on gene copy number estimates. The goal was to assess if individuals from the coastal and montane populations clustered separately as seen with the SNP data (Branco *et al.* 2015; Branco *et al.* 2017). To this end, we used matrices of estimates of per-individual gene copy numbers in 250 bp windows across all samples using the Bedtools software (Quinlan and Hall 2010; Quinlan 2014). We compiled four distance matrices by filtering the raw matrix in different ways. One matrix included all CNVs of coding DNA sequence (CDS) regions, a second included only the top 1% differentiated CDSs. We also filtered the matrices based on the number of copies: a third matrix only included regions that had at least one ‘gain’, and a fourth matrix included only regions that had at least one zero value. All distance matrices and PCoAs were generated using the *ape* package (Paradis and Schliep 2018) in R (R Core Team 2014). To compare montane and coastal CNV size and numbers we used a Wilcoxon rank sum test. Additionally, to visualize how similar individuals are to one another, we used the distance matrix used in the PCoA of the all the genes with CNVs to build a cluster-based tree using default settings the R package *ape* (Paradis and Schliep 2018). We were then able to compare our clustering tree with NJ tree based on SNPs from (Branco *et al.* 2015).

### Gene ontology enrichment analyses of the top 1% diverged CNVs

To assess whether the top CNV differentiated genes were enriched in genes involved in salt tolerance, we conducted a gene ontology (GO) enrichment analysis on the top 1% diverged CNVs between the two *S. brevipes* populations. We used GO terms based on the annotated reference genome of *S. brevipes* (Branco *et al.* 2015) and used the App ClueGO (Kirilovsky *et al.* 2009) in Cytoscape (Shannon *et al.* 2003) to identify significant GO terms and describe gene functions. We established significance of GO term enrichment by implementing the two-sided hypergeometric test and Benjamini–Hochberg corrected p-values using the ClueGO statistics.

### Genes of interest related to salt homeostasis

To identify genes related to salt tolerance we used the *Suillus brevipes* reference genome (Branco *et al.* 2015) housed in the MycoCosm database (Grigoriev *et al.* 2013). We searched the database for key words ‘Na^+^’ and ‘sodium’. Gene copy numbers were recovered using Bedtools (Quinlan 2014), and we built heatmaps depicting gene copy numbers in R (R Core Team 2014) using gplots (Warnes *et al.* 2009) and Rcolorbrewer (Neuwirth 2014).

We performed Fisher’s exact tests (R Core Team 2014) to investigate whether there was a significant difference in salt homeostasis-related gene copy number between populations. For each individual, copy numbers of selected genes were scored as a gain, absence, or neutral based on the reference genome. Fisher exact tests were performed with an expected odds ratio of 1:1 and a confidence interval of 95%. We used a Bonferroni correction for multiple testing. All code is available at https://github.com/abazzical/sbrevCNV.

### Data availability

 All raw data were taken from previous studies and are publicly available (Branco *et al.* 2015; Branco *et al.* 2017). All scripts for analyses are presented on https://github.com/abazzical/sbrevCNV. Supplemental material available at figshare: https://doi.org/10.25387/g3.13082483.

## Results

### Gene CNVs do not reflect SNP pattern in Suillus brevipes

**Figure 1:**
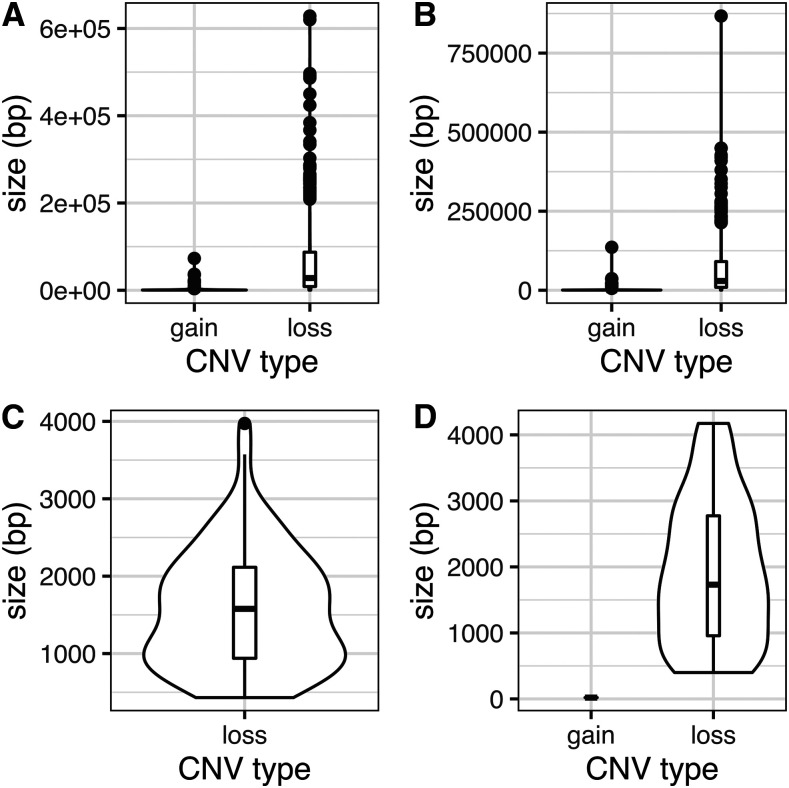
*Suillus brevipes* copy number variant sizes (bp) for the whole genome and for genes predicted to be involved in salt tolerance. A and B are sizes across the whole genome, C and D are sizes for predicted genes. A and C are the montane population and B and D are the coastal population.

Contrary to previous SNP results showing clear population differentiation (Branco *et al*. 2015), there were no significant differences in CNV number or CNV size between montane and coastal populations (Wilcoxon rank sum test *P* > 0.05). The vast majority of CNVs were losses across the whole genome (Table 1). Close to 10% of CNVs were located in predicted genes across all isolates. Consistent with findings in other fungi (Hartmann and Croll 2017), we found much fewer copy number gains compared to losses both across the genome and in predicted genes. The losses were on average much larger sections of the genome compared to the size of the duplications/multiplications (Table 1, Figure 1).

**Table 1:** Copy-number variants numbers and average size across all individuals for the whole genome and only regions predicted as genes

CNVs		Total # across individuals		Mean size across individuals (bp)	
		Montane	Coastal	Montane	Coastal
Whole genome	gains	89	95	3662.9	4810.5
Whole genome	losses	607	620	62852.4	64685.6
Predicted genes	gains	0	1	—	20
Predicted genes	losses	73	76	1556.7	1926.5

**Figure 2:**
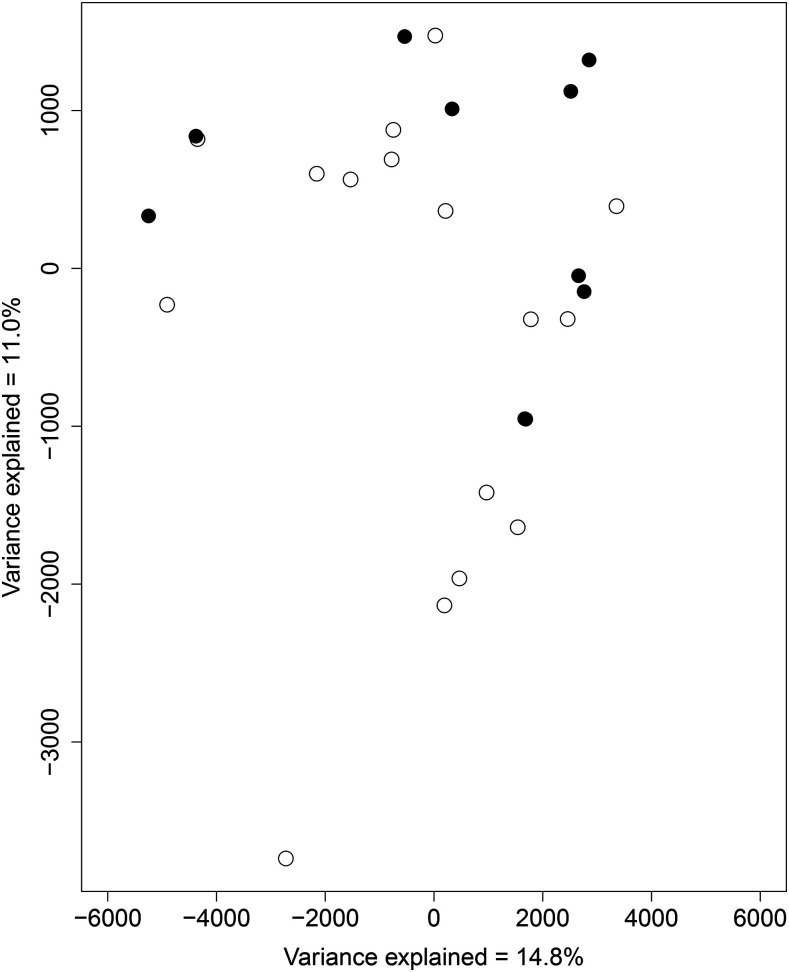
Gene copy number variants do not track *Suillus brevipes* population structure. Principal Coordinates Analysis of whole-genome copy number variants for *S. brevipes* individuals from coastal (full circles) and montane (empty circles) populations in California.

We also found CNVs do not show the same pattern as SNPs and failed to recover population structure across coastal and montane individuals of *S. brevipes* in California. This result was clear from both the whole genome CNV PCoA (Figure 2) and a cluster analysis (Fig S4). Neither analyses showed differentiation between the two populations as the individuals did not group based on their population of origin. We found variation in the number of copies of genes in different individuals both spread across the genome and in the salt tolerance related genes (see section below). When looking at only at regions showing a ‘gain’ or an ‘absence’ we found the ordinations to be indistinguishable which suggests that there is very little overall difference in gene losses and duplications (Fig S5 and S6).

### The top differentiated CNV regions are not enriched in genes related to salt homeostasis

**Figure 3:**
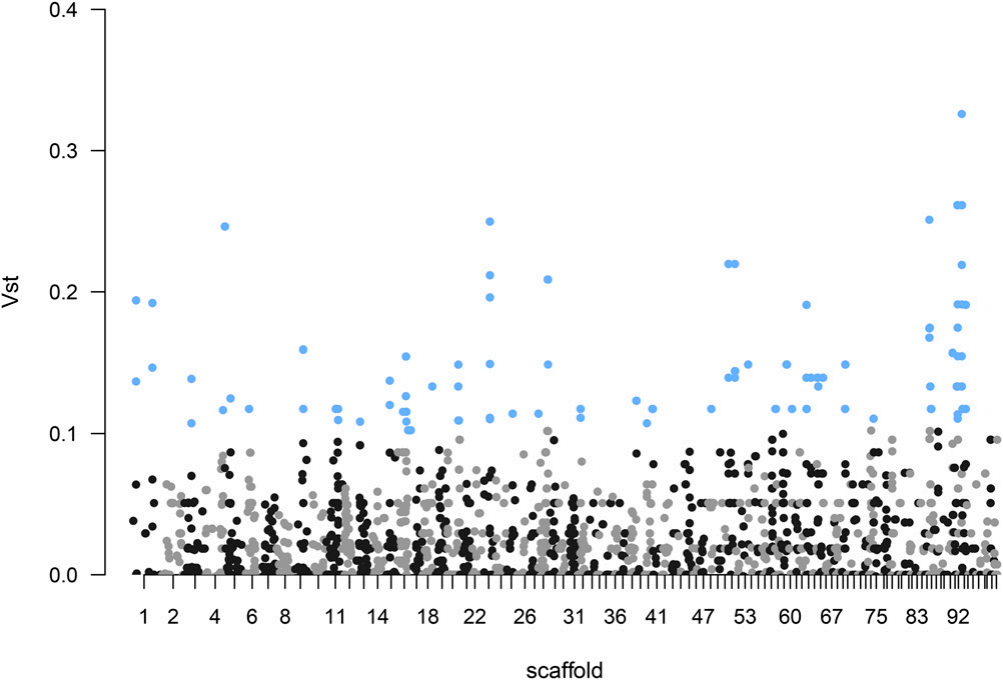
*Suillus brevipes* coastal/montane genomic divergence based on V_ST_ values (difference in variance of copy numbers between populations (Redon *et al.* 2006). The analysis was performed on 250 bp windows of the largest reference genome 100 scaffolds. Dots in blue represent the top 1% of the V_ST_ values and are enriched for ubiquitin.

Gene copy number variation was spread across the genome, with some regions showing much more differentiation compared to others (Figure 3). The PCoA based on the top 1% CNV differentiated genes showed a the coastal population clustered in a tight group which could be explained by stronger selection or recent bottleneck of the coastal population reported in (Branco *et al.* 2015) (Fig. S1). The top 1% 250bp windows overlapped with 37,537 annotations from the JGI reference genome and comprised 16,472 ‘CDS’, 18,362 ‘exon’, 1,347 ‘stop codon’, and 1,355 ‘start codon’ regions. Contradicting our expectations, the top 1% of the CNVs were not enriched in genes known to be related to salt tolerance. The gene ontology enrichment analysis on top 1% diverged genes revealed ‘ubiquitin’ as the sole significantly represented GO term (Table S1). Ubiquitin is present across eukaryotes and regulates proteins in the cell and it is unclear how this gene function relates to environmental or salt adaptation in the two *S. brevipes* populations (Hershko and Ciechanover 1998).

### Absence of copy-number differentiation in salt-tolerance specific genes between Suillus brevipes coastal and montane populations

**Figure 4:**
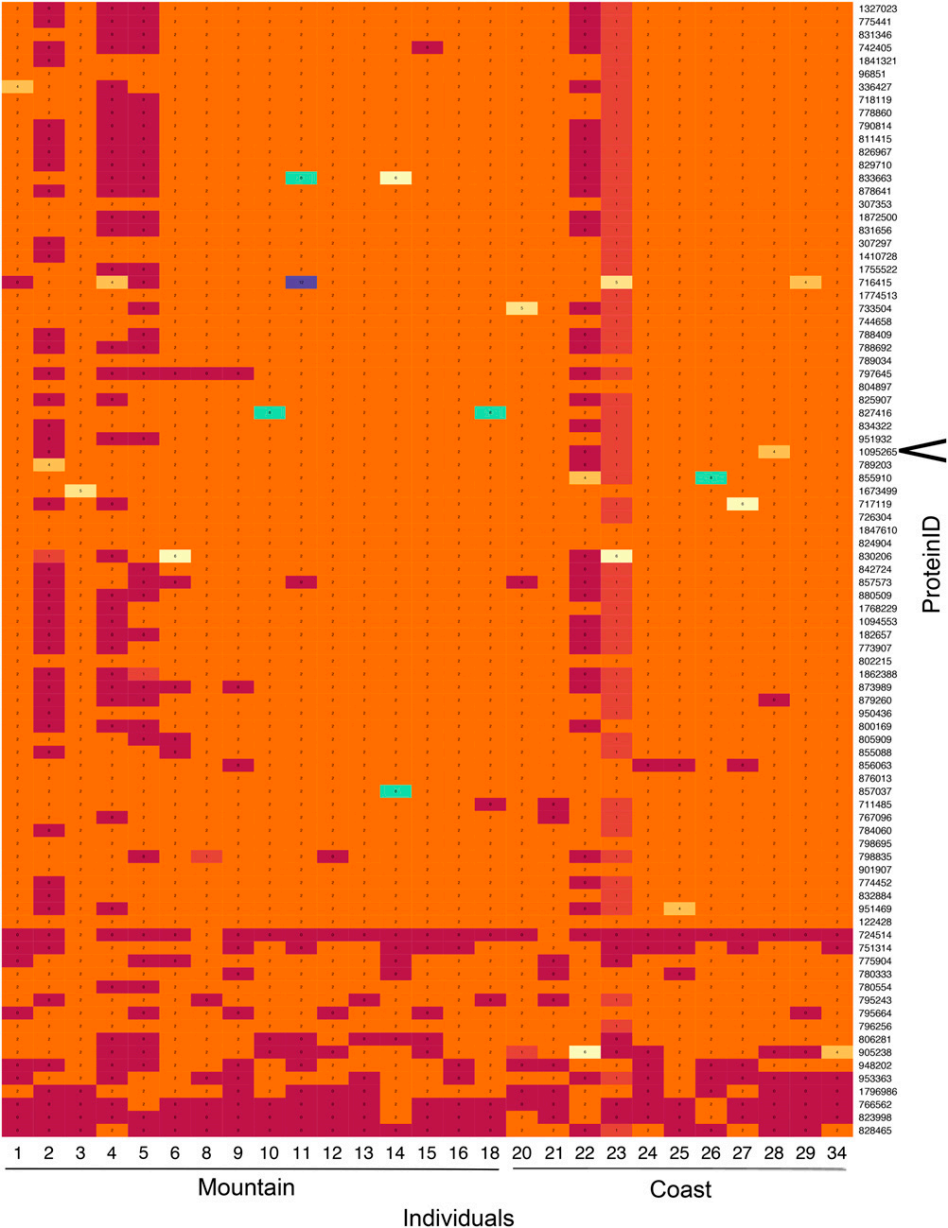
Gene copy number variation in *Suillus brevipes* genes involved in salt homeostasis. The heatmap shows gene copy number variation across 83 genes involved. The color scale from burgundy to blues going through yellows corresponds to numbers from ‘0’ (burgundy) to ‘12’ (blue). (Individuals 1-18 belong to the montane population and 20-34 to the coastal population). The *Nha-*like gene is indicated with an arrow ‘<’. The protein IDs of each gene are listed on the right-hand side of the plot.

We found 83 genes involved in salt homeostasis across the *S. brevipes* genome, including ‘sodium/calcium exchanger proteins’, ‘Na^+^/dicarboxylate, Na^+^/tricarboxylate and phosphate transporters’, and ‘Sodium/hydrogen exchanger family’. Based on Fisher’s Exact tests, none of these genes showed significant differences in CNVs between coastal and montane populations (Table S2), suggesting salt homeostasis genes are not under selection for different copy numbers in the two populations. However, as shown in the heatmap in Figure 4, there is variation in copy number across individuals of both populations.

The salt-related gene identified in Branco *et al.* (2015) (*Nha-1*-like gene; ProteinID 1095265) showed a range of copy numbers across individuals (Figure 4). However, there was no consistent pattern between the two populations, indicating absence of selection on the *Nha-1*-like gene copy number.

## Discussion

We found CNVs did not recover patterns of population structure and selection previously documented in *Suillus brevipes*. This species is known to be genetically differentiated across coastal and montane California and to show genomic signatures of local adaption to high soil salinity (Branco *et al.* 2015). Contrary to our expectations, we found no evidence of copy-number variation differentiation both across the genome and in salt tolerance related genes. However, individuals in both populations showed CNVs across their genomes which is consistent with copy-number variation in the ribosomal internal transcribed spacer for individuals of the same lichenized fungal species (Bradshaw *et al.* 2020).

Given the previous findings in *S. brevipes*, we hypothesized CNVs involved in salt homeostasis to be under selection, particularly the *Nha-1* like gene that was previously documented to be under positive selection. Individuals within a population share demographic history that impacts both SNP and gene copy number variation, making it is reasonable to expect both types of genetic variation display similar patterns of population structure and selection. In fact, such examples are well documented in human populations both across the whole genome (Jakobsson *et al.* 2008) and in specific genes associated with innate immune function (Ballana *et al.* 2007). There are also cases of congruence between SNP and CNV patterns in fungi, including in the plant pathogen *Zymoseptoria tritici*, where gene CNVs and SNP divergence show the same worldwide population structure (Hartmann and Croll 2017; Zhao and Gibbons 2018).

Both biological and technical factors likely contributed for the absence of concordance between CNVs and SNPs in *S. brevipes*, including recent population divergence, weak selection, and a highly fragmented reference genome. Despite being clearly distinct and isolated by discontinuous pine host presence in the California Central Valley, the coastal and montane *S. brevipes* populations share a fair amount of ancestral genetic variation indicating a rather recent split (Branco *et al.*, 2015) that might have not allowed for CNV differentiation to establish yet. In addition, weak natural selection can also potentially contribute to the absence of CNV signal. Even though there is clear evidence of local adaptation in *S. brevipes* from California (Branco *et al.* 2015, 2017), the previously detected salt tolerance genomic signature was restricted to a single region including one gene known to be involved in salt homeostasis, suggesting soil salinity might not be a very strong selective pressure for this species. The coastal and montane *S. brevipes* populations were sampled from locations with distinct soil chemistries (Peay *et al.* 2010) and the absence of genetic diversity in the *Nha-1* like gene in the coastal population was a good indication of soil salinity being a selective pressure for *S. brevipes* (Branco *et al.* 2015). However, it is possible that the coastal Californian soil salt content is not sufficiently high to lead to genetic signatures of adaptation on CNVs. Conversely, selection on salt-related CNVs could also be weak because the coastal variant of the sodium transport gene detected by Branco *et al.* (2015) is sufficient in providing an advantage in coastal environments. Finally, *S. brevipes* is not a model organism and has limited available genomic resources that could have prevented recovering a stronger CNV signal. The reference genome for this species is highly fragmented (assembled in 1550 scaffolds) and its annotation incomplete and based on sequence similarity-based gene predictions (Kuo *et al.* 2014). These resources allow for unveiling considerable aspects of *S. brevipes* biology but might have prevented the detection of key genes involved in environmental adaptation. We investigated ∼52% of the genome (included in the 100 largest scaffolds) and there might be adaptive CNVs related to salt tolerance in the remainder of the genome that remained undetected.

In conclusion, our results complement findings from previous studies on environmental adaptation in the mycorrhizal fungus *S. brevipes* (Branco *et al.* 2017; Branco *et al.* 2015) and more broadly contribute a previously unreported scenario of CNVs not reflecting patterns of population structure or selection displayed by SNP variation.

## References

[bib1] Apse M. P., Aharon G. S., Snedden W. A., and Blumwald E., 1999 Salt Tolerance Conferred by Overexpression of a Vacuolar Na+/H+ Antiport in *Arabidopsis*. Science 285: 1256–1258. 10.1126/science.285.5431.1256 10455050

[bib2] Ballana E., González J. R., Bosch N., and Estivill X., 2007 Inter-population variability of *DEFA3* gene absence: correlation with haplotype structure and population variability. BMC Genomics 8: 14 10.1186/1471-2164-8-14 17214878 PMC1779775

[bib3] Barillot E., Schleiermacher G., Janoueix-Lerosey I., Cappo J., Bleakley K., 2011 Control-FREEC: a tool for assessing copy number and allelic content using next-generation sequencing data. Bioinformatics 28: 423–425. 22155870 10.1093/bioinformatics/btr670 PMC3268243

[bib4] Bazzicalupo A. L., Ruytinx J., Ke Y.-H., Coninx L., Colpaert J. V., 2020 Fungal heavy metal adaptation through single nucleotide polymorphisms and copy-number variation. Mol. Ecol. 00: 1–13. 10.1111/mec.15618 32866320

[bib5] Bradshaw M., Grewe F., Thomas A., Harrison C. H., Lindgren H., 2020 Characterizing the ribosomal tandem repeat and its utility as a DNA barcode in lichen-forming fungi. BMC Evol. Biol. 20: 2 10.1186/s12862-019-1571-4 31906844 PMC6945747

[bib6] Branco S., 2019 Fungal diversity from communities to genes. Fungal Biol. Rev. 33: 225–237. 10.1016/j.fbr.2019.06.003

[bib7] Branco S., Bi K., Liao H. L., Gladieux P., Badouin H., 2017 Continental‐level population differentiation and environmental adaptation in the mushroom *Suillus brevipes*. Mol. Ecol. 26: 2063–2076. 10.1111/mec.13892 27761941 PMC5392165

[bib8] Branco S., Gladieux P., Ellison C. E., Kuo A., LaButti K., 2015 Genetic isolation between two recently diverged populations of a symbiotic fungus. Mol. Ecol. 24: 2747–2758. 10.1111/mec.13132 25728665

[bib9] Cheeseman I. H., Miller B., Tan J. C., Tan A., Nair S., 2015 Population Structure Shapes Copy Number Variation in Malaria Parasites. Mol. Biol. Evol. 33: 603–620. 10.1093/molbev/msv282 26613787 PMC4760083

[bib10] Chen Z., Cheng C.-H. C., Zhang J., Cao L., Chen L., 2008 Transcriptomic and genomic evolution under constant cold in Antarctic notothenioid fish. Proc. Natl. Acad. Sci. USA 105: 12944–12949. 10.1073/pnas.0802432105 18753634 PMC2529033

[bib41] Coninx L., Smisdom N., Kohler A., Arnauts N., Ameloot M. *et al.*, 2019 SlZRT2 Encodes a ZIP Family Zn Transporter With Dual Localization in the Ectomycorrhizal Fungus *Suillus luteus*. Front. in Microbio. 10: 2251. 10.3389/fmicb.2019.02251 PMC6797856 31681189

[bib11] Conrad D. F., Pinto D., Redon R., Feuk L., Gokcumen O., 2010 Origins and functional impact of copy number variation in the human genome. Nature 464: 704–712. 10.1038/nature08516 19812545 PMC3330748

[bib12] Craciun A. R., Meyer C.-L., Chen J., Roosens N., De Groodt R., 2012 Variation in HMA4 gene copy number and expression among *Noccaea caerulescens* populations presenting different levels of Cd tolerance and accumulation. J. Exp. Bot. 63: 4179–4189. 10.1093/jxb/ers104 22581842

[bib13] Dhar R., Sägesser R., Weikert C., Yuan J., and Wagner A., 2011 Adaptation of *Saccharomyces cerevisiae* to saline stress through laboratory evolution. J. Evol. Biol. 24: 1135–1153. 10.1111/j.1420-9101.2011.02249.x 21375649

[bib14] Dorant, Y., H. Cayuela, K. Wellband, M. Laporte, Q. Rougemont *et al.*, 2020 Copy number variants outperform SNPs to reveal genotype-temperature association in a marine species. bioRxiv 10.1101/2020.01.28.923490 Preprint posted January 29, 2020. 32803780

[bib15] Ellison C. E., Hall C., Kowbel D., Welch J., Brem R. B., 2011 Population genomics and local adaptation in wild isolates of a model microbial eukaryote. Proc. Natl. Acad. Sci. USA 108: 2831–2836. 10.1073/pnas.1014971108 21282627 PMC3041088

[bib16] Gladieux P., Wilson B. A., Perraudeau F., Montoya L. A., Kowbel D., 2015 Genomic sequencing reveals historical, demographic and selective factors associated with the diversification of the fire‐associated fungus *Neurospora discreta*. Mol. Ecol. 24: 5657–5675. 10.1111/mec.13417 26453896

[bib17] Gordon S. P., Contreras-Moreira B., Woods D. P., Des Marais D. L., Burgess D., 2017 Extensive gene content variation in the Brachypodium distachyon pan-genome correlates with population structure. Nat. Commun. 8: 2184 10.1038/s41467-017-02292-8 29259172 PMC5736591

[bib18] Grigoriev I. V., Nikitin R., Haridas S., Kuo A., Ohm R., 2013 MycoCosm portal: gearing up for 1000 fungal genomes. Nucleic Acids Res. 42: D699–D704. 10.1093/nar/gkt1183 24297253 PMC3965089

[bib19] Gu W., Zhang F., and Lupski J. R., 2008 Mechanisms for human genomic rearrangements. PathoGenetics 1: 4 10.1186/1755-8417-1-4 19014668 PMC2583991

[bib20] Hartmann F. E., and Croll D., 2017 Distinct trajectories of massive recent gene gains and losses in populations of a microbial eukaryotic pathogen. Mol. Biol. Evol. 34: 2808–2822. 10.1093/molbev/msx208 28981698 PMC5850472

[bib21] Hershko A., and Ciechanover A., 1998 The Ubiquitin System. Annu. Rev. Biochem. 67: 425–479. 10.1146/annurev.biochem.67.1.425 9759494

[bib22] Huang S., Spielmeyer W., Lagudah E. S., and Munns R., 2008 Comparative mapping of *HKT* genes in wheat, barley, and rice, key determinants of Na+ transport, and salt tolerance. J. Exp. Bot. 59: 927–937. 10.1093/jxb/ern033 18325922

[bib23] Hull R. M., Cruz C., Jack C. V., and Houseley J., 2017 Environmental change drives accelerated adaptation through stimulated copy number variation. PLoS Biol. 15: e2001333 10.1371/journal.pbio.2001333 28654659 PMC5486974

[bib24] Jakobsson M., Scholz S. W., Scheet P., Gibbs J. R., VanLiere J. M., 2008 Genotype, haplotype and copy-number variation in worldwide human populations. Nature 451: 998–1003. 10.1038/nature06742 18288195

[bib25] Kirilovsky A., Mlecnik B., Pagès F., Bindea G., Hackl H., 2009 ClueGO: a Cytoscape plug-in to decipher functionally grouped gene ontology and pathway annotation networks. Bioinformatics 25: 1091–1093. 10.1093/bioinformatics/btp101 19237447 PMC2666812

[bib26] Kondrashov F. A., 2012 Gene duplication as a mechanism of genomic adaptation to a changing environment. Proc. Biol. Sci. 279: 5048–5057. 22977152 10.1098/rspb.2012.1108 PMC3497230

[bib27] Kuo A., Bushnell B., and Grigoriev I. V., 2014 Chapter One - Fungal Genomics: Sequencing and Annotation, pp. 1–52 in Advances in Botanical Research, edited by Martin F. M. Academic Press, Cambridge, MA.

[bib28] Lupski J. R., and Stankiewicz P., 2005 Genomic disorders: molecular mechanisms for rearrangements and conveyed phenotypes. PLoS Genet. 1: e49 10.1371/journal.pgen.0010049 16444292 PMC1352149

[bib29] Møller H. D., Parsons L., Jørgensen T. S., Botstein D., and Regenberg B., 2015 Extrachromosomal circular DNA is common in yeast. Proc. Natl. Acad. Sci. USA 112: E3114–E3122. 10.1073/pnas.1508825112 26038577 PMC4475933

[bib30] Nass R., Cunningham K. W., and Rao R., 1997 Intracellular Sequestration of Sodium by a Novel Na+/H+ Exchanger in Yeast Is Enhanced by Mutations in the Plasma Membrane H+-ATPase: INSIGHTS INTO MECHANISMS OF SODIUM TOLERANCE. J. Biol. Chem. 272: 26145–26152. 10.1074/jbc.272.42.26145 9334180

[bib31] Neuwirth E., 2014 RColorBrewer: ColorBrewer palettes. https://cran.ma.imperial.ac.uk/web/packages/RColorBrewer/RColorBrewer.pdf

[bib32] Oh D.-H., Dassanayake M., Bohnert H. J., and Cheeseman J. M., 2012 Life at the extreme: lessons from the genome. Genome Biol. 13: 241 10.1186/gb-2012-13-3-241 22390828 PMC3439964

[bib33] Paradis E., and Schliep K., 2018 ape 5.0: an environment for modern phylogenetics and evolutionary analyses in R. Bioinformatics 35: 526–528. 10.1093/bioinformatics/bty633 30016406

[bib34] Peay K. G., Garbelotto M., and Bruns T. D., 2010 Evidence of dispersal limitation in soil microorganisms: Isolation reduces species richness on mycorrhizal tree islands. Ecology 91: 3631–3640. 10.1890/09-2237.1 21302834

[bib35] Prior C., Potier S., Souciet J.-L., and Sychrova H., 1996 Characterization of the *NHA1* gene encoding a Na+/H+-antiporter of the yeast *Saccharomyces cerevisiae*. FEBS Lett. 387: 89–93. 10.1016/0014-5793(96)00470-X 8654575

[bib36] Quinlan A. R., 2014 BEDTools: the Swiss‐army tool for genome feature analysis. Curr. Protoc. Bioinformatics 47: 11.12.1–11.12.34. 10.1002/0471250953.bi1112s47 PMC4213956 25199790

[bib37] Quinlan A. R., and Hall I. M., 2010 BEDTools: a flexible suite of utilities for comparing genomic features. Bioinformatics 26: 841–842. 10.1093/bioinformatics/btq033 20110278 PMC2832824

[bib38] R Core Team, 2014 R: A language and environment for statistical computing, R Foundation for Statistical Computing, Vienna, Austria.

[bib39] Redon R., Ishikawa S., Fitch K. R., Feuk L., Perry G. H., 2006 Global variation in copy number in the human genome. Nature 444: 444–454. 10.1038/nature05329 17122850 PMC2669898

[bib40] Riehle M. M., Bennett A. F., and Long A. D., 2001 Genetic architecture of thermal adaptation in *Escherichia coli*. Proc. Natl. Acad. Sci. USA 98: 525–530. 10.1073/pnas.98.2.525 11149947 PMC14620

[bib42] Schrider D. R., and Hahn M. W., 1698 2010 Gene copy-number polymorphism in nature. Proceedings of the Royal Society B: Biological Sciences 277: 3213–3221. 10.1098/rspb.2010.1180 PMC2981937 20591863

[bib43] Shannon P., Markiel A., Ozier O., Baliga N. S., Wang J. T., 2003 Cytoscape: a software environment for integrated models of biomolecular interaction networks. Genome Res. 13: 2498–2504. 10.1101/gr.1239303 14597658 PMC403769

[bib44] Steenwyk J., and Rokas A., 2017 Extensive copy number variation in fermentation-related genes among *Saccharomyces cerevisiae* wine strains. G3 (Bethesda) 7: 1475–1485. 28292787 10.1534/g3.117.040105 PMC5427499

[bib45] Steenwyk J. L., and Rokas A., 2018 Copy number variation in fungi and its implications for wine yeast genetic diversity and adaptation. Front. Microbiol. 9: 288 10.3389/fmicb.2018.00288 29520259 PMC5826948

[bib46] Steenwyk J. L., Soghigian J. S., Perfect J. R., and Gibbons J. G., 2016 Copy number variation contributes to cryptic genetic variation in outbreak lineages of *Cryptococcus gattii* from the North American Pacific Northwest. BMC Genomics 17: 700 10.1186/s12864-016-3044-0 27590805 PMC5009542

[bib47] Talke I. N., Hanikenne M., and Krämer U., 2006 Zinc-Dependent Global Transcriptional Control, Transcriptional Deregulation, and Higher Gene Copy Number for Genes in Metal Homeostasis of the Hyperaccumulator *Arabidopsis halleri*. Plant Physiol. 142: 148–167. 10.1104/pp.105.076232 16844841 PMC1557598

[bib48] Warnes G. R., Bolker B., Bonebakker L., Gentleman R., Huber W., 2009 gplots: Various R programming tools for plotting data, pp. 1.

[bib49] Wu H.-J., Zhang Z., Wang J.-Y., Oh D.-H., Dassanayake M., 2012 Insights into salt tolerance from the genome of *Thellungiella salsuginea*. Proc. Natl. Acad. Sci. USA 109: 12219–12224. 10.1073/pnas.1209954109 22778405 PMC3409768

[bib50] Zhang Y., Hu Y., Wang X., Jiang Q., Zhao H., 2020 Population Structure, and Selection Signatures Underlying High-Altitude Adaptation Inferred From Genome-Wide Copy Number Variations in Chinese Indigenous Cattle. Front. Genet. 10: 1404 10.3389/fgene.2019.01404 32117428 PMC7033542

[bib51] Zhao S., and Gibbons J. G., 2018 A population genomic characterization of copy number variation in the opportunistic fungal pathogen Aspergillus fumigatus. PLoS One 13: e0201611 10.1371/journal.pone.0201611 30071059 PMC6072042

